# RETRACTED: Elalfy et al. ATT-Myc Transgenic Mouse Model and Gene Expression Identify Genotoxic and Non-Genotoxic Chemicals That Accelerating Liver Tumor Growth in Short-Term Toxicity. *Biomedicines* 2025, *13*, 743

**DOI:** 10.3390/biomedicines14092085

**Published:** 2026-09-16

**Authors:** Mahmoud Elalfy, Ahmed Jaafar Aljazzar, Mona G. Elhadidy

**Affiliations:** 1 Clinical Science Department, College of Veterinary Medicine, King Faisal University, Al-Ahsa 3959-36362, Saudi Arabia; 2 Pathology Department, College of Veterinary Medicine, King Faisal University, Al-Ahsa 3959-36362, Saudi Arabia; ajazzar@kfu.edu.sa; 3 Medical Physiology, Faculty of Medicine, Mansoura University, Mansoura City 35516, Egypt; dr_monagaber@mans.edu.eg; 4 Medical Physiology, Faculty of Medicine, Al-Baha University, Alaqiq 65779-7738, Saudi Arabia; mona.j@bu.edu.sa

The journal retracts and amends the authorship list of the article titled “ATT-Myc Transgenic Mouse Model and Gene Expression Identify Genotoxic and Non-Genotoxic Chemicals That Accelerating Liver Tumor Growth in Short-Term Toxicity” [1], cited above.

Following publication, concerns were brought to the attention of the publisher by an originally listed author regarding the integrity of several subfigures and the accuracy of the authorship list.

According to the journal’s standard procedures, the Editorial Office and Editorial Board conducted an investigation. This investigation identified duplications within Figures 1 and 5, including material overlapping with a previously published article [2], presenting different experimental conditions. The investigation further confirmed, with input from the Ombuds Office of Hannover Medical School, that the originally published authorship list and stated contributions were inaccurate. Jurgen Borlak was included as an author in the original submission without his consent and did not approve the publication. Although the authors cooperated with the investigation and provided supporting materials, the Editorial Board concluded that the concerns could not be satisfactorily resolved based on the available information. Consequently, the Editorial Board has lost confidence in the reliability of the reported findings and has decided to retract the article and amend its authorship, removing Jurgen Borlak from the authors’ list, in accordance with MDPI’s retraction policy.

This retraction was approved by the Editor-in-Chief of the journal *Biomedicines*.

The authors have been informed of this retraction and have indicated their disagreement with this decision.
